# DL_FFLUX: A Parallel, Quantum Chemical Topology Force Field

**DOI:** 10.1021/acs.jctc.1c00595

**Published:** 2021-10-07

**Authors:** Benjamin C. B. Symons, Michael K. Bane, Paul L. A. Popelier

**Affiliations:** † Manchester Institute of Biotechnology (MIB), 131 Princess Street, Manchester M1 7DN, Great Britain; ‡ Department of Chemistry, University of Manchester, Oxford Road, Manchester M13 9PL, Great Britain; § High End Compute LTD, 23 Welby Street, Manchester M13 0EL, Great Britainhttps://highendcompute.co.uk; ∥ Department of Computing and Mathematics, Manchester Metropolitan University, Manchester M15 6BH, Great Britain

## Abstract

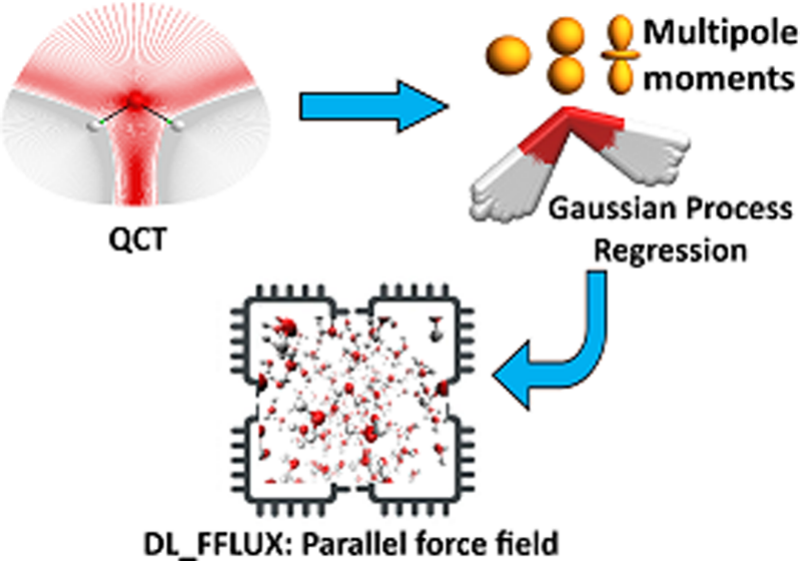

DL_FFLUX is a force field based on quantum chemical topology that can perform molecular dynamics for flexible molecules endowed with polarizable atomic multipole moments (up to hexadecapole). Using the machine learning method kriging (aka Gaussian process regression), DL_FFLUX has access to atomic properties (energy, charge, dipole moment, etc.) with quantum mechanical accuracy. Newly optimized and parallelized using domain decomposition Message Passing Interface (MPI), DL_FFLUX is now able to deliver this rigorous methodology at scale while still in reasonable time frames. DL_FFLUX is delivered as an add-on to the widely distributed molecular dynamics code DL_POLY 4.08. For the systems studied here (10^3^–10^5^ atoms), DL_FFLUX is shown to add minimal computational cost to the standard DL_POLY package. In fact, the optimization of the electrostatics in DL_FFLUX means that, when high-rank multipole moments are enabled, DL_FFLUX is up to 1.25× faster than standard DL_POLY. The parallel DL_FFLUX preserves the quality of the scaling of MPI implementation in standard DL_POLY. For the first time, it is feasible to use the full capability of DL_FFLUX to study systems that are large enough to be of real-world interest. For example, a fully flexible, high-rank polarized (up to and including quadrupole moments) 1 ns simulation of a system of 10 125 atoms (3375 water molecules) takes 30 h (wall time) on 18 cores.

## 1 Introduction

A computational chemistry code needs to compete along two axes: accuracy and speed. Generally, an improvement in one comes at the expense of the other. Classical force fields are well known to be fast but often lack accuracy.^1,2^ Ab initio methods typically achieve greater accuracy than classical force fields, but this accuracy comes at a high computational cost. This cost limits ab initio methods to relatively small systems or short simulation time scales. Thus, a holy grail of computational chemistry is finding a method,^3−17^ often involving machine learning nowadays, that is fast and accurate, the equivalent of “having one’s cake and eat it”.

DL_FFLUX is a force field that aims to perform accurate molecular dynamics (MD) calculations without sacrificing speed too much. The accuracy of the FFLUX methodology has been demonstrated at various levels of analysis from single molecules^18−21^ to small clusters of molecules^22^ and even ions.^23^ However, before the advances presented in the current paper, the prohibitive computational cost of DL_FFLUX made “bulk” simulations impossible. The current work describes, in detail, a step change toward making DL_FFLUX a practical but more reliable alternative to well-known and popular force fields.

The accomplishment of DL_FFLUX up to now can be attributed to its rigorous theoretical foundation^24^ and design from scratch. At the heart of DL_FFLUX is the quantum topological atom offered by the quantum theory of atoms in molecules (QTAIM),^25^ which led to the energy partitioning scheme called interacting quantum atoms (IQA).^26^ Both are part of quantum chemical topology (QCT), a term coined^27^ in 2003 to refer to the collection of approaches^28^ sharing the idea of a (gradient) vector field partitioning a quantum mechanical function. If this function is the electron density, then the (quantum) topological atom emerges.

The computational cost associated with QCT is mitigated in two ways. First, the results of QCT calculations are used to train machine learning models, specifically kriging or Gaussian process regression^29,30^ method of machine learning. After compute-intensive training, these kriging models can then quickly predict atomic energies and multipole moments, using only the nuclear coordinates of the given atom’s environment. The predictions occur in real time, during the course of an MD simulation, at considerably less cost than performing quantum mechanical calculations. Moreover, kriging needs fewer data to reach a given accuracy compared^11,31^ compared to neural nets. It should also be mentioned here that polarization is taken care of without the need for on-the-fly dipole moment iterations using polarizabilities. Second, DL_FFLUX is now parallelized with Message Passing Interface (MPI),^32^ so it can take advantage of high-performance computing hardware.

In summary, DL_FFLUX can perform MD simulations with fully flexible molecules and polarizable atomic multipole moments (up to hexadecapole moment). Molecular flexibility and polarization are achieved without recourse to the usual means of harmonic potentials,^33^ Drude oscillators,^34^ or other methods.^35,36^ However, in DL_FFLUX, predicted (intramolecular) potential energy surfaces and atomic multipole moments handle molecular flexibility, polarization, and intramolecular charge transfer. All this information is captured by the kriging models trained on an effective paucity of computationally expensive QCT data. The advance presented here is that DL_FFLUX is currently optimized and parallelized such that QCT data are now available during an MD simulation at a relatively low extra cost.

## 2 Theoretical Background

### 2.1 QTAIM

The gradient of the electron density, ∇ρ(*r⃗*), defines a topological atom as a naturally emerging subspace. Each atom comprises an attractor (the nucleus) and a particular portion of the total electron density. Each nucleus attracts a bundle of trajectories of the gradient of the electron density, which are commonly referred to as gradient paths. The subspace spanned by this bundle is the topological atom. The boundaries of an atom are interatomic surfaces, which are surfaces of zero-flux of ∇ρ(*r⃗*). In other words, each atom inside a molecule is bounded by surfaces that obey eq 1
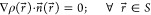
1

**Figure 1:**
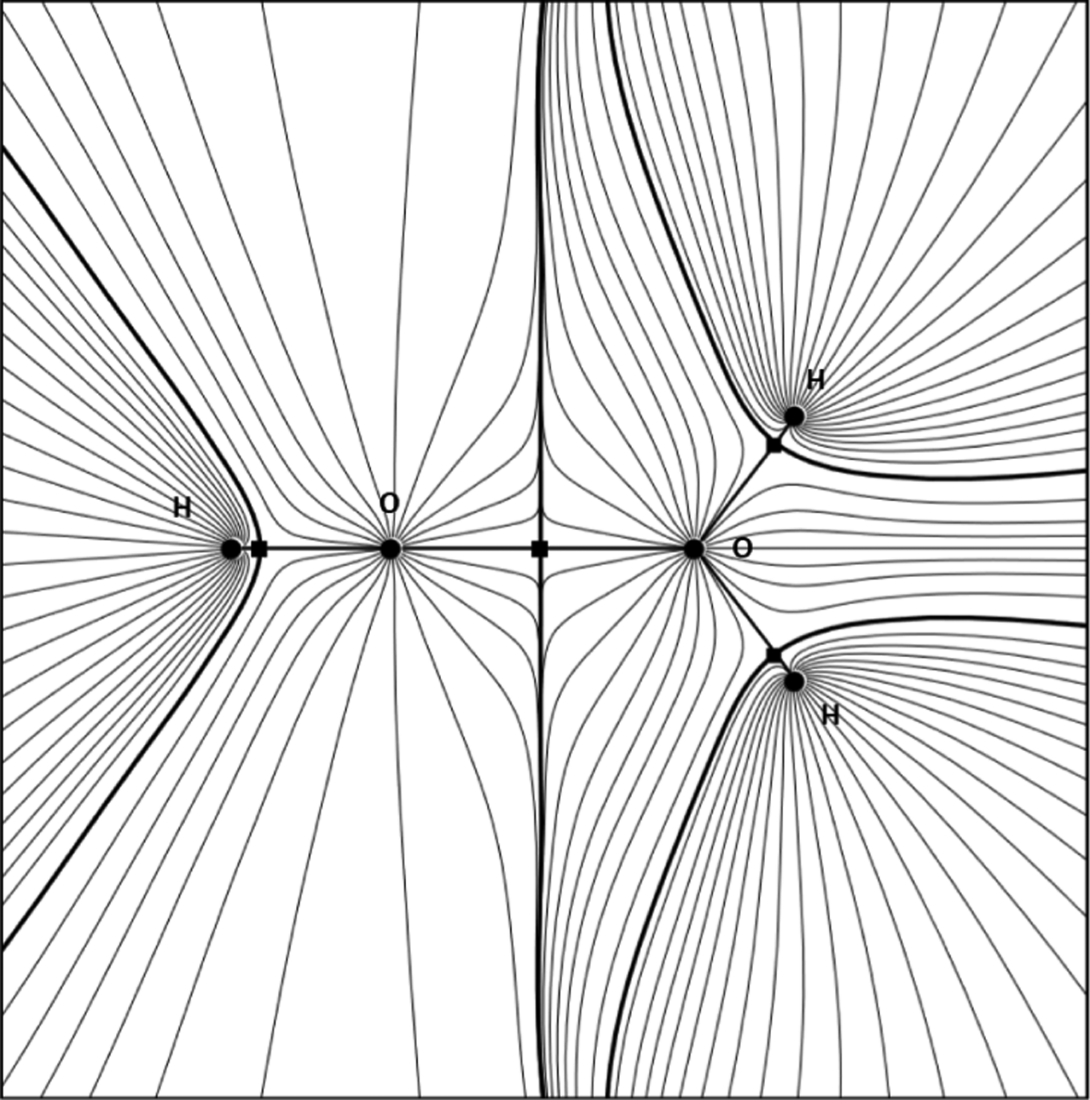
Gradient paths for a water dimer. The circles represent nuclei (attractors). Thick black lines are interatomic surfaces, and the squares that lie on these lines are so-called bond critical points. The second hydrogen of the left water is perpendicular to the plotting plane and not shown.

The constraint in eq 1 defines a surface that is not crossed by any gradient paths, which is clear from Figure 1, where a water dimer complex illustrates various topological objects.

Where an atom is not bordered by another atom, its electron density extends out to infinity. However, to have a practical boundary to an atom at the edge of a molecule, the atom can be capped by an arbitrary constant electron density surface. We also note that topological atoms are space-filling: they leave no gaps and do not overlap.

### 2.2 IQA

This relatively recent scheme decomposes atomic energies in a chemically meaningful way,^37^ without the problems^38^ of older energy decomposition analyses such as natural energy decomposition analysis (NEDA).^39^ Inspired by early calculations^40^ of the electrostatic energy between topological atoms, IQA extends QTAIM’s original virial-based energy partitioning scheme such that atomic energies are also valid for nonequilibrium geometries. IQA is a reference-free and exhaustive partitioning scheme, two properties shown to be favorable.^41^ The IQA energy decomposition is achieved by partitioning the one- and two-electron density matrices. However, the derivation^26^ is not discussed here, only the results.

Atomic IQA energies can be broken down into intra-atomic and interatomic contributions
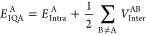
2

Both terms in eq 2 can be broken down further as follows
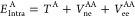
3
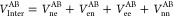
4

The superscripts in eqs 3 and 4 indicate the two atoms that are interacting with each other, while the subscripts indicate the type of interaction. The intra-atomic energy in eq 3 is composed of the kinetic energy, *T*^A^, and nuclear–electron and electron–electron interactions. The interatomic term in eq 4 also contains the nuclear–nuclear interaction. Note that the interatomic electron–nuclear interaction needs to be accounted for both ways: the nucleus of one atom must be paired with the electrons of the other atom and vice versa. DL_FFLUX does not see the finer details outlined in eqs 2–4. Rather, DL_FFLUX works with *E*_IQA_^A^ values, which is the only level of resolution necessary for MD.

### 2.3 Gaussian Process Regression or Kriging

Kriging is a machine learning technique that takes inputs (features) and maps them to a single output. In this case, the outputs are IQA energies and atomic multipole moments. The features for a given atom are geometric and defined in an atomic local frame (ALF). Three atoms are required to construct the axes of the ALF. The features of atoms that are not used to construct the ALF are composed of spherical polar coordinates defined with respect to this ALF. Each atom is the center of its own ALF. Full details of this framework can be found in our previous work.^19^ The choice of a local frame is significant as it means that all multipole moments are invariant with respect to global rotations and translations.

Kriging makes predictions based on some training set containing *N*_train_ training points. The training of kriging models is a topic unto itself^20^ and is not covered here as it is not relevant. Predictions of a quantity of interest, *Ŷ*^A^, relating to (topological) atom A, are made according to
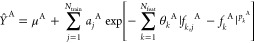
5where μ^A^ is the average value of the output for all training points, *a*_*j*_^A^ is the “weight” of the *j*th training point, and θ_*k*_^A^ and *p*_*k*_^A^ are hyperparameters optimized during training (although the latter are typically set to “2” without much loss of accuracy). The exponent’s argument contains *N*_feat_-dimensional vectors of features where the number of features is equal to the dimensionality of the system. Equation 5 essentially finds the correlation between a set of (previously unseen) input features *f⃗*^A^ and the features of the training data *f⃗*_*j*_^A^ and predicts based on this correlation. The form of eq 5 follows that, if a set of inputs is “far away” from any of the training points, the exponent’s argument approaches zero and the predicted output returns to the average, μ^A^. This is a potential weakness if the feature space has been trained for a narrow sample of configuration space that does not cover the geometries encountered during a simulation.

For the purposes of DL_FFLUX, *Ŷ*^A^ is either *Ê*_IQA_^A^ or one of the multipole moments, {*Q̂*_*l*,*m*_}. In the case of the IQA energy, DL_FFLUX also calculates the forces^42^ resulting from these energies. These are the intramodel forces, which for our purposes are synonymous with intramolecular energies. However, it is possible to have models describe and predict quantum system than single molecules; molecular complexes can also be treated by this methodology. The *i*th component of the force on atom B is calculated as
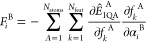
6

Equation 6 is the sum of derivatives of the IQA energy of every atom A with respect to the global Cartesian coordinates of atom B, α_*i*_^B^. This derivative is obtained via a chain rule involving the features d*f*_*k*_^A^. Note that eq 6 replaces the use of traditional harmonic bond and angle potentials in DL_FFLUX, a hallmark of DL_FFLUX’s fresh approach to force field design.

### 2.4 Electrostatics

Electrostatics in DL_FFLUX is worthy of a brief discussion. Short-range electrostatics are captured by the kriging models and are wrapped up inside *E*_IQA_^A^. Short range for our purposes means intramolecular. On the other hand, long-range electrostatics (intermolecular) is then calculated using smooth particle mesh Ewald^43^ with polarizable moments predicted by kriging models. The rank of the electrostatic interaction is denoted *L*′. This quantity refers to the highest-rank multipole moments present in a given simulation. For example, *L*′ = 2 means that monopole (*l* = 0), dipole moments (*l* = 1) and quadrupole moments (*l* = 2) are present and that monopole–monopole, monopole–dipole, monopole–quadrupole, dipole–dipole, dipole–quadrupole, and quadrupole–quadrupole interactions are computed. At present, FFLUX supports moments up to and including hexadecapole moments (hence *L*′ = 4). It has been shown previously that point charge only does not suffice for the convergence of long-range electrostatic interactions in water.^44^ The need for high-rank multipolar electrostatics has been observed in the small protein crambin as well.^45^

Overall and in summary, DL_FFLUX is a truly new force field, which is much closer to the underlying quantum reality. DL_FFLUX “sees the electrons” and exploits a parameter-free definition of an atom inside a system. Using machine learning, DL_FFLUX learns how atomic energies, charges, and multipole moments vary with the surrounding atoms’ geometry. As such it captures all polarization and many-body effects, as well as charge transfer, in one streamlined scheme. The approach avoids perturbation theory and thus benefits from a clear treatment of short-range interactions. Moreover, DL_FFLUX breaks free from the rigid-body constraints of advanced polarizable force fields. The well-defined atom at the heart of DL_FFLUX enables physics-based machine learning. It uses kriging instead of neural nets, thereby reducing the training data size. Finally, we point out DL_FFLUX’s modularity: each energy term represents only the physical phenomenon it describes and nothing else e.g., the electrostatic energy is well defined. If this contribution is improved, then the other nonelectrostatic terms will not be affected. Hence, DL_FFLUX is systematically improvable; energy reliability does not depend on fortuitous cancellation or compensation between different force field terms, as is the case with classic force fields.

### 2.5 Domain Decomposition (DD)

**Figure 2:**
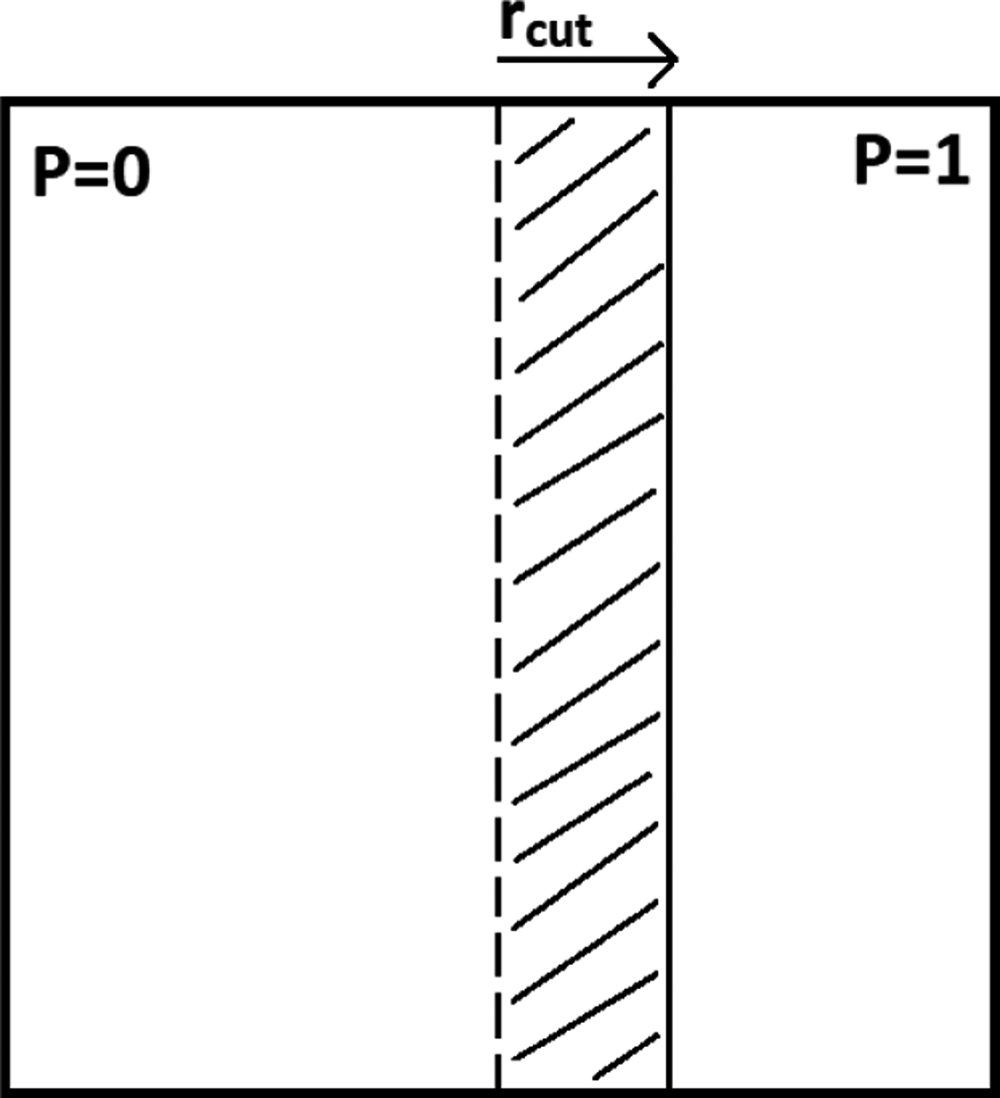
Two-dimensional simulation cell split into two domains. The dashed line represents the divide between the domains. The halo of *P* = 0 is shown by the shaded area.

There are several approaches one can take to implementing MPI in a given program. Discussed here is the domain decomposition (DD) method.^46^ This method was chosen for DL_FFLUX primarily because DD is already implemented in DL_POLY 4.08. The domain decomposition method is relatively simple in principle. In DD MPI, the simulation cell is partitioned into smaller cells called domains such that each of the *N*_p_ processes computes some subset of the total system in parallel. Figure 2 demonstrates this concept for a simple two-dimensional (2D) system: the space is split in half as there are two MPI processes, where *P* = 0 and 1 denote the first and second processes, respectively.

In the ideal case, *N*_p_ domains would lead to a speed-up given by
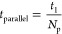
7where *t*_1_ is the time on one single process (i.e., serial). However, in practice, eq 7 is complicated by the need for communications between domains. It is also possible that not all of the code is parallelized and thus a more realistic equation for speed-up is
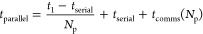
8where *t*_serial_ and *t*_comms_(*N*_p_), respectively, represent the time spent in serial code and in MPI communications, the latter being some possibly nontrivial function of *N*_p_. Serial code is a hard limiting factor on speed-up. For example, if 10% of the runtime is spent in serial code, then the maximum possible speed-up is a factor of 10 according to Amdahl’s law. This situation can only be improved by parallelizing any serial code.

MPI communications are typically necessary when calculations in a given domain require information from neighboring domain(s) or for synchronization purposes, among other reasons. In MD simulations, any pairwise (or many-body) interactions between particles located on different domains will require some degree of communication. In practice, some particle *i*, located in a given domain, will interact with all particles *j* that satisfy the condition *r*_*ij*_ < *r*_cut_, where *r*_*ij*_ = |*r⃗*_*ij*_| is the distance between particles *i* and *j*, and *r*_cut_ is the cutoff radius. Any particle *j* that satisfies the cutoff condition but is not in the same domain as *i* will require a communication between the processes associated with the domains of *j* and *i*. Information such as *j*’s coordinates and multipole moments will have to be communicated. Note that communication is also required in the DD method when a particle crosses the boundary between domains during a simulation.

The way to deal with these communications is to construct a so-called halo for each domain, which is shown in Figure 2. A domain’s halo contains all of the information it needs about neighboring domains. The data required by a process can then be split into two parts: local and halo. The local part is the information about the particles that reside in a process’ domain and the halo is all of the extra information needed to compute any interactions between particles in different domains. A halo need only extend a distance *r*_cut_ beyond a domain’s borders because interactions between particles separated by a larger distance are not computed. In practice, a halo typically extends slightly further than *r*_cut_.

It is evident from Figure 2 that the smaller the *r*_cut_, the smaller the halo. A useful ratio is given by eq 9
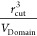
9where *V*_Domain_ is the volume of a domain. As this ratio decreases, the proportion of particles in a given domain that require communications also decreases. This ratio can be decreased by reducing *r*_cut_ but this may reduce the accuracy of the simulation. Instead, the size of a domain can be increased. This can be done by increasing the overall system size with a fixed *N*_p_ or by reducing *N*_p_ for a given system size. For a given system, there will come a point when increasing *N*_p_ starts to slow down the code due to the increased cost of communications (note the optimal *N*_p_ is highly system-dependent). Another important factor to consider is load balancing. If the density of the system is highly inhomogeneous, then different processes may have significantly different amounts of computation to carry out, i.e., the computational load is imbalanced. If this is the case, then some processes will have to wait for others to finish, which negatively impacts parallel performance. Provided the density is relatively homogeneous, this issue should not occur when using domain decomposition.

**Figure 3:**
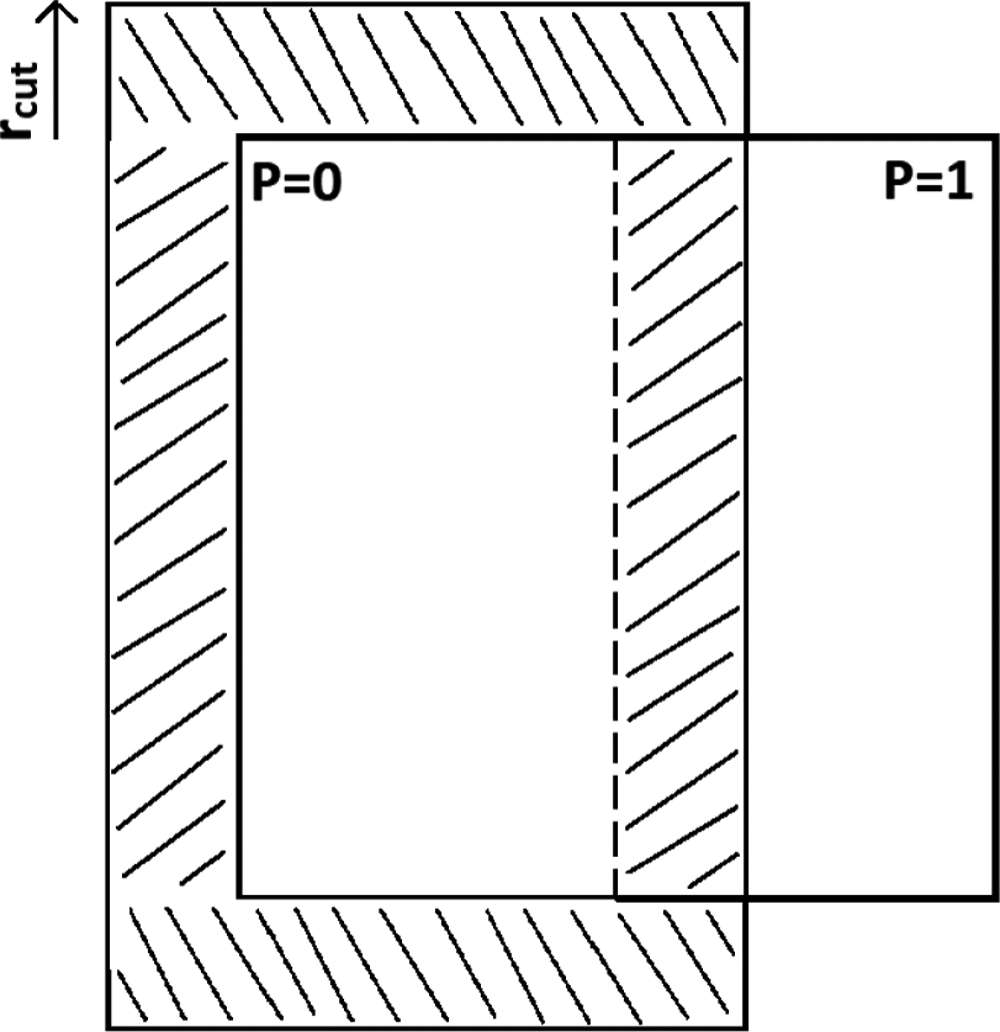
Two-dimensional simulation cell split into two domains. The full halo of *P* = 0 is shown by the shaded area, accounting for PBCs.

It is worth noting that the DD method must be extended if it is combined with periodic boundary conditions (PBCs). In this case, the DD halo must be combined with a PBC halo. Instead of containing just particles from neighboring domains the halo may also include image particles from neighboring image cells. Figure 3 shows the full halo of the domain associated with *P* = 0 in the case of MPI and PBCs. The portion of the halo that extends beyond the main simulation cell is the part that contains image particles.

## 3 Code Structure

### 3.1 Overview

**Figure 4:**
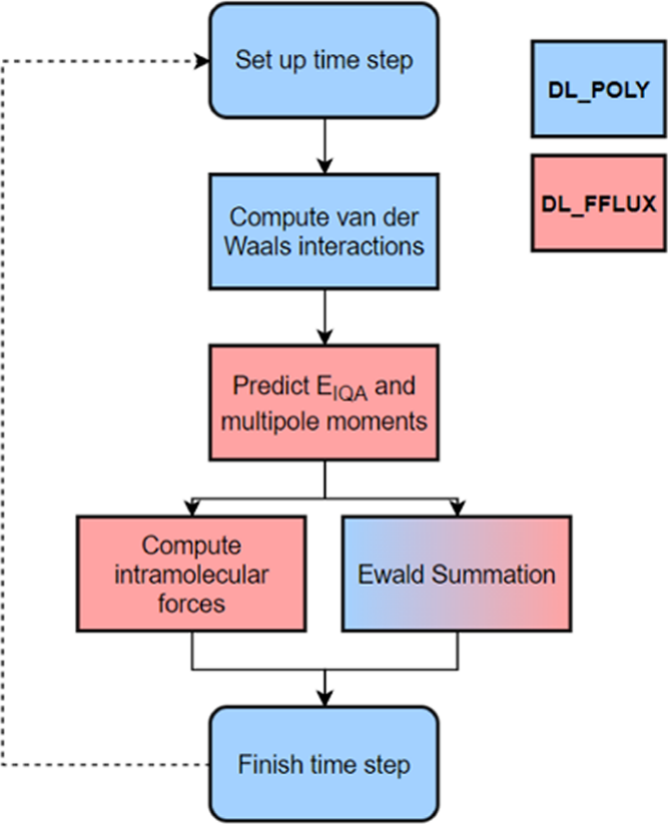
Flow diagram demonstrating the stages of a typical DL_FFLUX MD time step.

DL_FFLUX is a FORTRAN90 code, written as a modular attachment to the code DL_POLY 4.08.^47,48^ Note that some bug fixes from DL_POLY 4.09 have been integrated into DL_FFLUX. DL_FFLUX implements the FFLUX methodology and comprises 17 subroutines as well as some changes to the DL_POLY source code to interface the two. DL_FFLUX options can be set in the same way as typical DL_POLY options using the CONTROL file. DL_POLY provides the framework for the MD part of the code, i.e., the parts of an MD simulation that are not changed by DL_FFLUX. The role of DL_FFLUX can be split into two parts. The first part makes predictions using kriging models that have been trained prior to running the MD simulation. In every time step, atomic IQA energies and multipole moments (potentially up to hexadecapole moment) are predicted. The multipole moments are fed into Ewald summation subroutines, DL_FFLUX specific, as well as heavily modified versions of the DL_POLY Ewald subroutines. The second part computes the intramolecular forces that result from the gradient of the predicted *E*_IQA_ potential energy surface. Figure 4 demonstrates a typical time step.

The flow diagram in Figure 4 does not imply that the computation of intramolecular forces and the Ewald summation is carried out simultaneously but rather that the DL_FFLUX predictions feed into both sections of the code. However, the two routines are independent and so they could run concurrently, in principle. Concurrent computation is not explored here but will be the subject of future work. Note that the Ewald summation is a blend of colors as it involves a mixture of DL_FFLUX, and modified DL_POLY, subroutines.

### 3.2 MPI

#### 3.2.1 DL_POLY

DL_POLY already implements the DD method of MPI. However, the challenge remained of suitably modifying the DL_FFLUX code to be compatible with DL_POLY’s existing implementation. For a given time step in a DL_POLY MD simulation, there are several places where calls to MPI routines are present. Most importantly, at the start of a time step, the halos must be constructed, and at the end of a time step, any particles crossing a domain boundary must be relocated. MPI calls are also needed for the fast Fourier transforms required in the reciprocal space part of the Ewald summation as well as in I/O routines.

Full details of DL_POLY’s parallelization strategy can be found in the user manual^49^ as well as in ref (50). However, a brief overview is given here. At the beginning of a time step, each domain calculates which of its particles each of its neighbors will require in their halos. The information on these particles is packed into a buffer and sent to the relevant neighboring domain. Each domain can then build up its own halo from the data it receives from its neighbors. The cost of these communications is minimized by sending data in three waves, that is, in the ±*x*, ±*y*, and ±*z* directions. Note that the order of these waves does not matter.^51^ It is important to mention here that DL_POLY actually divides domains further into link cells. Each link cell has side lengths, *l*, which is as close as possible to (but always greater than) *r*_cut_. Link cells enable the construction of a link list for every atom. The link list is a list of every atom inside a given atom’s cutoff radius. The main benefit of the link list method over, for example, the Verlet neighbor list relates to computational cost. The Verlet method computes all pairs of distances, i.e., *N*(*N* – 1), to construct the list of all pairs of atoms that interact. However, in the link list approach, one divides space into so-called link cells approximately of size *R*_cut_ such that, when constructing the list of atoms that a given atom can interact with, one only has to inspect the neighboring link cells (26 neighbors in three-dimensional (3D)). Computing all pairwise distances for the Verlet method is an *O*(*N*^2^) operation, whereas the link list method turns out to be an approximately *O*(*N*) calculation. However, the link cell method only becomes more efficient^51^ than the Verlet neighbor list when the number of link cells per dimension is greater than 3 as it is only when this condition is met that the method computes fewer than all pairwise distances.

The final aspect of the DL_POLY DD implementation to consider is the choice of indexing. Each process has a subset of all of the relevant property arrays such as positions, velocities, forces, etc. Approximately, the subsets will be of size natms/*N*_p_. These arrays can either retain global indices or be indexed locally.

In the global case, the indices of atoms do not change. However, all loops must be altered so that each process operates only on the indices corresponding to atoms that reside in its domain. DL_POLY uses local indexing instead. In this case, the arrays on each process are indexed from 1 to *n*_last_, which is the number of local atoms plus the number of halo atoms on a given process. The variable natms, which stores the total number of atoms in the system in the serial case, is redefined on each process to be the number of local atoms in that domain. This means that loops in the main code do not need to be altered when going from serial to MPI, thus preserving a single source code. However, this complicates matters slightly as there needs to be a mapping from local to global indices. It is also useful to have the reverse mapping. This mapping is nontrivial, especially in the case of PBCs because a global atom can appear multiple times in a domain’s halo, which means that there may not be a unique mapping from a global index to a single local index. This issue is solved in DL_POLY by storing a list of the local indices and a local list of global indices that have been sorted. A binary search algorithm is then used to search the sorted list.

#### 3.2.2 DL_FFLUX

**Figure 5:**
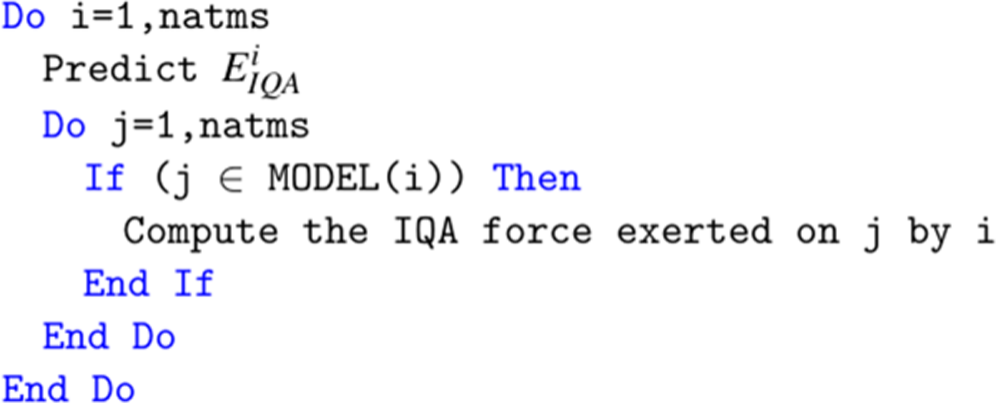
Pseudo code for DL_FFLUX IQA energy and force calculation loop before modification.

One of the main parts of DL_FFLUX is the IQA energy prediction and intramolecular force calculation loop. This loop had to be modified to work with DD MPI. The original structure of the loop is shown in Figure 5. There is a loop over all natms local atoms. The IQA energy of atom *i*, *E*_IQA_^*i*^ is predicted and then the force that is exerted by *i* on all other atoms is computed in a second, interior loop. The force calculation is subject to the constraint that atom *j*, the atom that atom *i* exerts a force on, is part of the same kriging model as *i*. For our current purposes, the term model is synonymous with molecule, i.e., an atom can exert an IQA force on another atom only if both atoms belong to the same molecule.

**Figure 6:**
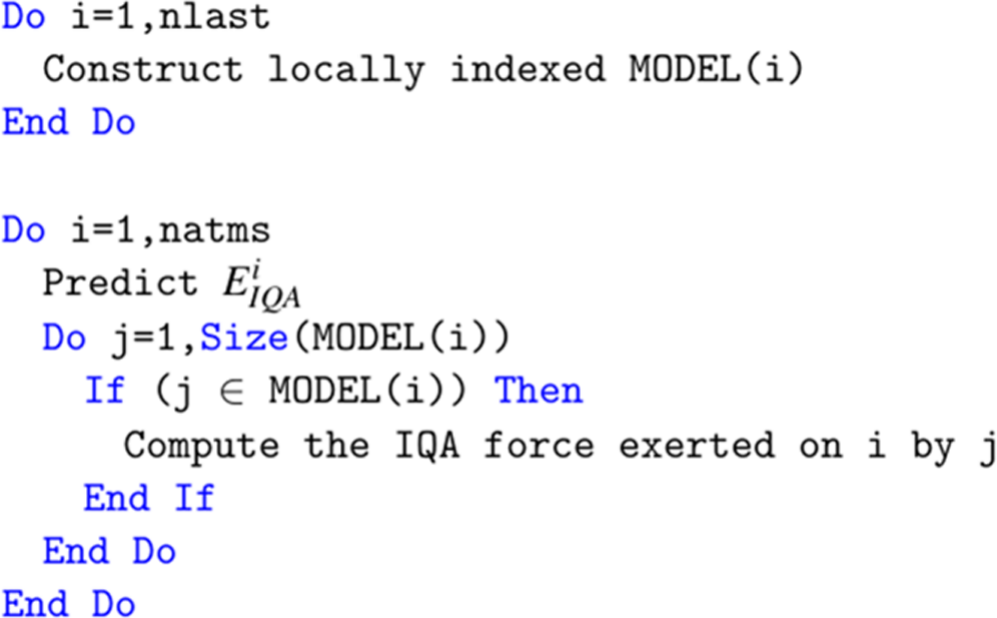
Pseudo code for DL_FFLUX IQA energy and force calculation loop after modification.

The routine has been changed in several ways as demonstrated in Figure 6. Prior to any computation of energies and forces, the globally indexed model arrays that keep track of which atoms share a kriging model need to be converted to local indices. Note that Figures 5 and 6 show only the MODEL array for the sake of simplicity, but, in reality, there are two arrays needed for this purpose. If this were not done, then local indices would be fed into these arrays and incorrect global indices would be returned. In other words, DL_FFLUX would not keep proper track of which atoms belong to the same model leading to incorrect predictions and resulting forces. Note that while only the first “natms” entries (the local part) of the MODEL array are needed for the force calculation in Figure 6, the rest of the array is required at a later point and so it is necessary to convert to local indices for the full array.

For the DD method to work properly, a given process must compute all forces exerted on its local atoms by all local and halo atoms. The original code in Figure 5 did not do this as both loops were indexed from 1 to natms. Given the outer loop corresponded to the atom exerting the force, only the forces exerted on local atoms by local atoms would be computed. This would pose an issue for any molecules that do not reside entirely in a single domain. To resolve this issue, the inner loop now occurs explicitly over all model atoms which, thanks to the localization of the model arrays, could be local atoms or part of the halo.

**Figure 7:**
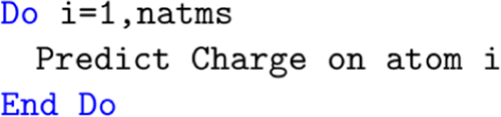
Original charge prediction loop.

The other major change to the DL_FFLUX code concerns the prediction of the multipole moments. In the case of point charges, this is done in a routine called fflux_ewald. This routine predicts atomic charges at every time step and then feeds them into DL_FFLUX and modified versions of DL_POLY’s Ewald routines to compute the long-range electrostatics. In the serial case, moments are predicted in a simple loop, as shown in Figure 7.

When PBCs are also included, there is a slight complication as the image atoms also need charges. However, these are all copies of main cell atoms, which means that the charges have already been predicted. As such, the image atoms in the PBC halo are just assigned the same charges as their main cell counterparts without requiring extra predictions.

**Figure 8:**
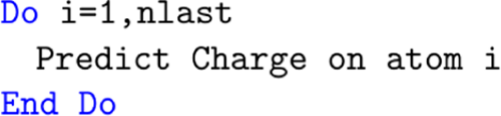
Modified charge prediction loop.

In the case of MPI, the modification to the loop is very simple and is shown in Figure 8. The only difference here is that the loop now runs to nlast rather than natms. This means that charges are predicted for all local and halo atoms. This is correct but involves some redundant work. The halo consists of main cell atoms that reside in a neighboring domain, which do require new charge predictions. However, it also consists of image atoms, which may not require new charge predictions. The impact of this redundant work is discussed in Section 4.

In the case of multipole moments of higher rank than point charge, the construction of the multipole component of the halo is done differently. Rather than re-predicting the halo moments as in the case of point charges, the halo moments are instead filled in by MPI communications. This is done in exactly the same way as the rest of the halo is constructed. The only difference is that it is done mid time step. Arguably this is a less elegant solution than constructing every aspect of the halo at the start of a time step. However, it does not appear to impact performance. In the future, this could, with some considerable restructuring of code, be integrated into the DL_POLY halo construction. However, this would involve considerable alteration of DL_POLY routines thus compromising the modularity of DL_FFLUX.

## 4 Results

Four systems were considered, which consist of water boxes of different sizes: 5184 atoms; 10 125 atoms; 46 875 atoms, and 107 811 atoms. The dimensions of the boxes were chosen to give approximately correct liquid densities at 300 K. All simulations were carried out within the NVT ensemble using the Nosé–Hoover thermostat with a 1 fs time step and a 9 Å cutoff radius unless stated otherwise. Note that the simulations also require nonbonded potentials, for example, Lennard-Jones or Buckingham. For this work, Lennard-Jones potentials were used for OO and OH with the following parameters: ε_OO_ = 0.753, σ_OO_ = 3.23, and ε_OH_ = 0.1063, σ_OH_ = 2.165 (units are kJ/mol and Å for ε and σ, respectively). An MPI process always binds to a single processor core. These four test cases provide a reasonable range of system sizes. Unless stated otherwise, all benchmarks were performed on a single node comprising two Intel Xeon Gold 6152 “Skylake” chips, each with 22 cores and a nominal clock speed of 2.10 GHz. The node has 768 Gb of RAM.

The machine learning models used for these simulations were trained on calculations of single molecules carried out at the B3LYP/aug-cc-pVTZ level of theory. The geometries used to train the models (as well as for the test set) were obtained using ab initio molecular dynamics with the program CP2K.^52^ Thanks to recent advances in our training procedure using adaptive sampling,^20^ the models are very compact (just 32 training points) while maintaining accuracy with a total root-mean-square error (RMSE) of 1.3 kJ/mol on a test set of 500 points (the total RMSE is the sum of the RMSE for each atom). The multipole moments are also well predicted. For example, for the same test set, the RMSE of the oxygen charge is 0.0007 *e*. Note that further improvements in training since this work was done mean that the latest water model has 59 training points with an RMSE of 0.17 kJ/mol for the total energy. The time to train the models was on the order of a few hours (which is a one-off cost).

The focus of this paper is purely the performance of the code in terms of timings and parallel efficiency. The quality of the simulations as assessed by bulk properties is not discussed as it will be the subject of an upcoming paper.

The Supporting Information (SI) provides extra information such as Figures S1–S4, which examine how the runtime scales as a function of system size for a given value of the number of processes *N*_p_. Figures S1–S4 show the relationships for *N*_p_ = 1, 2, 4, and 8, respectively, with a range extending to more than 100 000 atoms. Second, Table S1 shows how the code scales with cutoff radius (for the 107 811-atom system).

### 4.1 Optimization

Alongside parallelization, the DL_FFLUX code has been extensively rewritten to be faster and more memory-efficient when running in serial. This section compares the serial un-optimized (original) and optimized codes, that is, without any MPI. The optimized code now uses up to a factor of 10^4^ less memory for the systems studied (the larger the system, the larger the saving). The effects of optimization on the runtime are best demonstrated by comparing timings and profiles of the serial code before and after optimization. Time and memory constraints of the un-optimized code^22^ mean that only the smallest system (5184 atoms) could be studied prior to optimization. More detail about the optimization is given in Section S6 in the SI.

**Figure 9:**
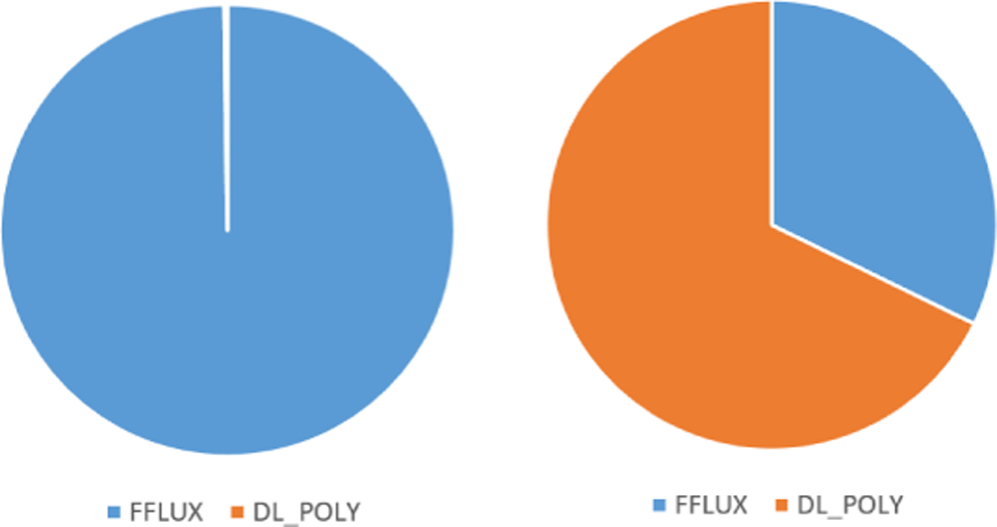
Profiles of the un-optimized (left) and optimized (right) code broken down into DL_FFLUX and DL_POLY contributions. The charts are not to scale, the right-hand-side chart represents a computation that is ∼75 000× faster than the left-hand-side chart.

The profiles in Figure 9 are completely different. The un-optimized profile is dominated entirely by DL_FFLUX, taking 99.8% of the runtime. After optimization, DL_FFLUX takes a considerably more modest 32.2% of the runtime.

The timings in Table 1 also demonstrate large speed-ups when running in serial. Results are reported in time taken for a 1 ns simulation with 1 fs time steps, and a 9 Å electrostatic and van der Waals cutoff radius. Note that the original DL_FFLUX code uses a different definition of the rank of electrostatic interaction. Rather than *L*′, the original code uses *L* = *l*_A_ + *l*_B_ + 1, where *l*_A_ and *l*_B_ are the rank of the multipoles on atoms *A* and *B*, respectively. In other words, in the (*l*_A_, *l*_B_) matrix of possible interacting multipole moments, *L* refers to a triangle while *L*′ refers to a square. This means that, for *L* = 2, for example, dipole moments are enabled but only the monopole–monopole and dipole–monopole (or dipole–monopole) interactions are computed. Note that dipole–dipole interactions are not computed because then *L* would be 1 + 1 + 1 = 3. However, for *L*′, we can say that the closest equivalent *L*′ value to *L* = 2 is *L*′ = 1, which computes also the dipole–dipole interaction. As such, when comparing the old and new DL_FFLUX it is important to recognize that the new code is calculating many more electrostatic interactions as *L* (or *L*′) becomes larger. Figure S5 in the SI examines the computational cost of increasing *L*′ in more detail.

**Table 1:** Comparison of Time Taken to Perform a 1 ns Simulation of the 5184-Atom Water Box Using the Original (Un-Optimized) and Optimized Codes^a^

	un-optimized	optimized	speed-up
*L* = 1/*L*′ = 0	250 years	29.0 h	75 569×
*L* = 2/*L*′ = 1	1709 years	4.7 days	132 811×
*L* = 3/*L*′ = 2	6573 years	6.5 days	369 352×

a Note that the un-optimized timings are estimates based on single time step simulations.

### 4.2 Strong Scaling

Strong scaling is the scaling of runtime with the number of processes (note that Section 1 in the SI shows weak scaling, i.e., scaling of runtime with problem size at fixed *N*_p_). All four systems were run at each of the three levels of electrostatic interaction (*L*′ = 0, 1, and 2) at various values of *N*_p_ to study the strong scaling.

**Figure 10:**
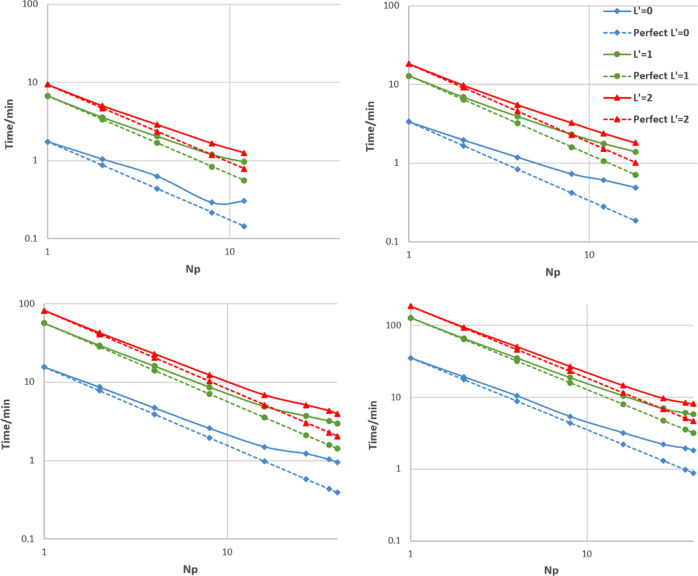
Strong scaling, both actual and perfect, on log–log axes for all four water boxes: (top left) 5184 atoms in a (38 Å)^3^ box; (top right) 10 125 atoms in a (47 Å)^3^ box; (bottom left) 46 875 atoms in a (78 Å)^3^ box; and (bottom right) 107 811 atoms in a (103.5 Å)^3^ box.

The strong scaling data for all four systems are shown in Figure 10. It is evident that all systems behave in a broadly similar way although the overall quality of the scaling tends to be better as system size increases. Larger systems are also able to utilize more processes (as the cell can be divided into more domains) and so often achieve a greater overall speed-up. From now on, data will not necessarily be shown for all systems to avoid repetition. Any data not given in the main paper can be found in the SI. We will now focus on the largest system (107 811 atoms) to analyze the scaling in more depth. Any discussion generalizes to all four systems unless stated otherwise.

**Figure 11:**
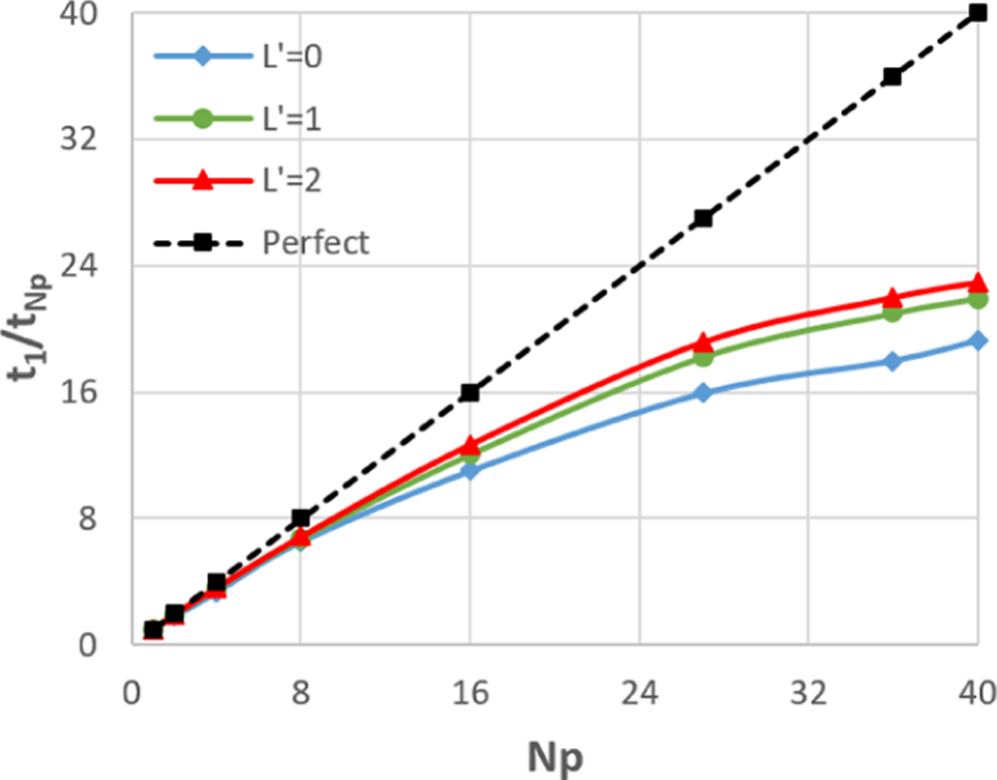
Speed-up of the whole code relative to serial for the 107 811-atom water box.

Figure 11 provides a different measure of the quality of the scaling, plotting *t*_1_/*t*_*N*_p__ (the time when run on a single process compared to that on *N*_p_ processes) rather than time itself. This ratio is the factor by which the code has been sped up by going from serial to *N*_p_ processes. In the perfect case, this will simply be equal to *N*_p_.

It is clear from Figure 11 that *L*′ = 0 scales worse than *L*′ = 1 or 2. This is generally the case for all four systems as can be seen in Figures 10 and S6–S8, which report on the remaining three systems. Profiling revealed the culprit is the aforementioned redundant prediction work being done in the case of *L*′ = 0, which is avoided when implementing MPI communications for higher-rank multipole moments. In the case of *L*′ = 0, the predictions scale poorly with *N*_p_, whereas, for *L*′ = 2, they scale considerably better.

**Figure 12:**
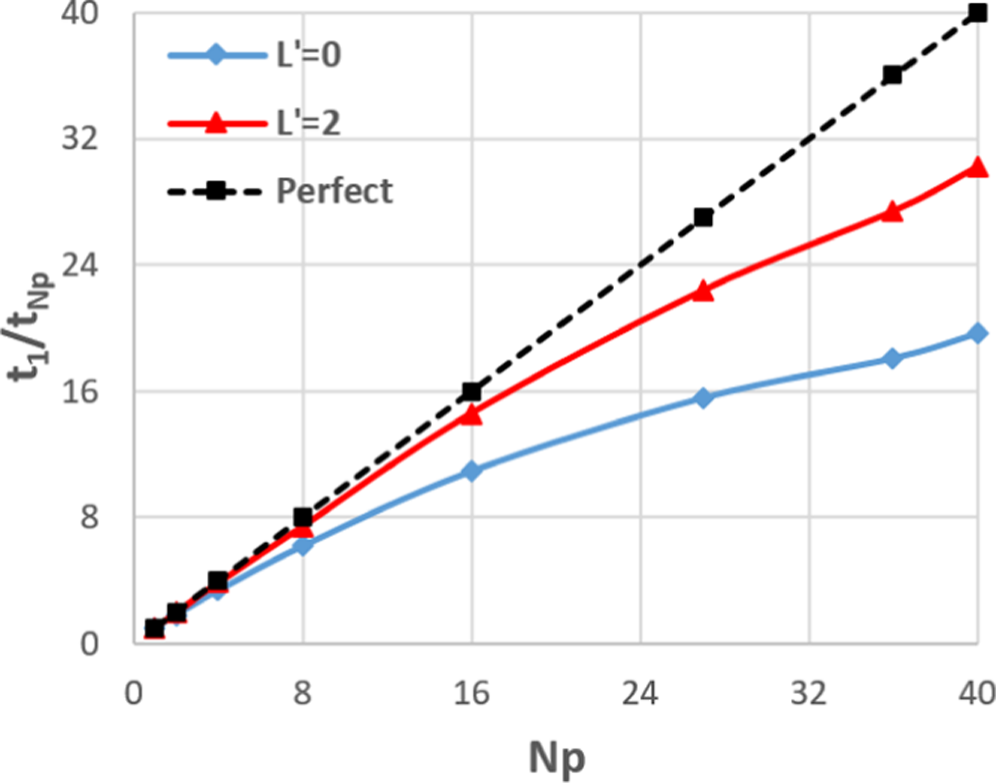
Speed-up of the DL_FFLUX prediction routine only relative to serial for the 107 811-atom water box.

The poor scaling of predictions for *L*′ = 0 shown in Figure 12 is exacerbated by the fact that the predictions take on average 26% of the runtime, while for *L*′ = 2, the predictions take on average 9% of the runtime. Note *L*′ = 1 is not considered here as initial profiles showed performance essentially identical to *L*′ = 2. There is more actual work to be done by the prediction routine for *L*′ = 2 because there are considerably more multipole moments to predict per atom. However, the removal of the redundant work and the massively increased cost of the electrostatics routines means that predictions take up a relatively small fraction of the overall work. This analysis suggests that it would be beneficial to remove the redundant work associated with the point charge halo in the same way as is done for higher-rank multipole moments. This will be the subject of future development of DL_FFLUX.

**Figure 13:**
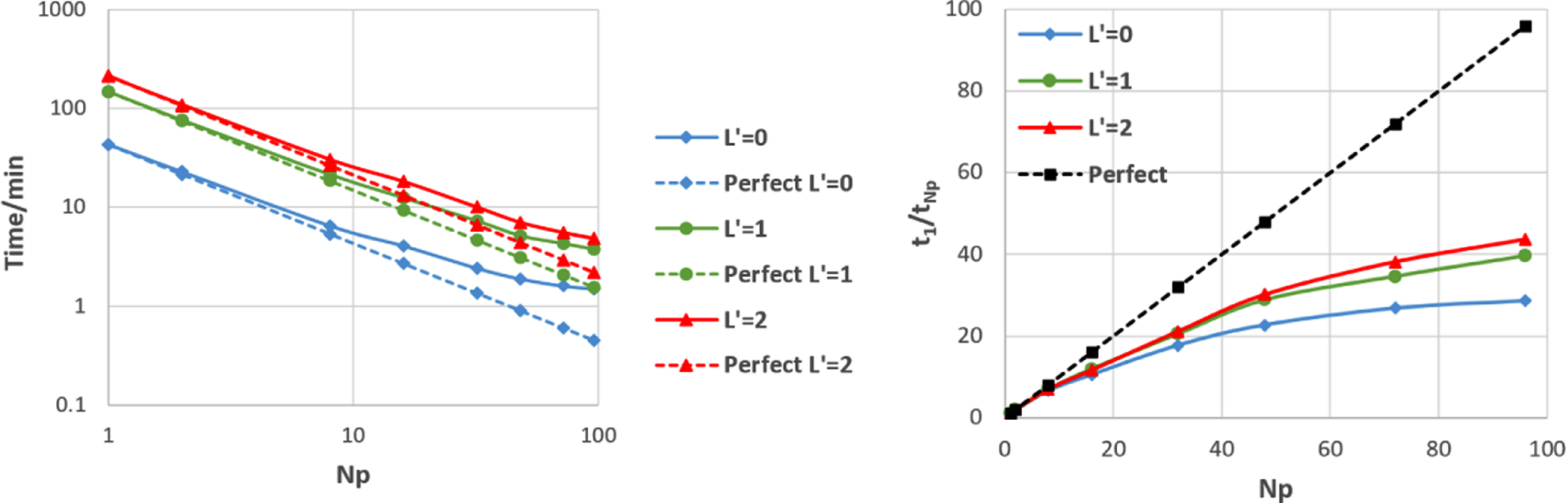
Strong scaling on multiple nodes for the 107 811-atom system. Log–log scaling (left) and speed-up relative to serial (right).

Until now, all tests have been confined to a single node. However, the performance of MPI across multiple nodes is a key metric. As such, the largest system (107 811 atoms) was studied on a set of nodes, each with two 12-core Intel Xeon E5-2690 v3 “Haswell” chips with a nominal clock speed of 2.6 GHz and 128 Gb RAM per node as well as Mellanox InfiniBand. All timings in Figure 13 (apart from serial) have been obtained on at least two nodes, e.g., *N*_p_ = 2 means one process per node, where each process still corresponds to a single core. The scaling in the right-hand-side graph of Figure 13 is comparable to that in Figure 11 up to *N*_p_ = 40 (the maximum in Figure 11) despite the processes being split over multiple nodes in Figure 13. This shows that the internode communications in DL_FFLUX are not overly costly and do not prohibit scaling across multiple nodes, at least for small numbers of nodes. For *L*′ = 2, the best speed-up increases from a factor of 23× (Figure 11) to a factor of 44× using four nodes.

### 4.3 Computational Cost of DL_FFLUX

Perhaps the most relevant question pertaining to DL_FFLUX’s performance is how much extra computational cost the methodology incurs over more traditional MD techniques (note that we do not compare to ab initio methods here as the test systems used are too large). DL_FFLUX is based on DL_POLY, so a comparison between the two codes is a natural one to draw. There are multiple ways to explore this question. First, a “direct” comparison can be drawn between “plain vanilla” DL_POLY with a commonly used water potential and DL_FFLUX. To this end, we performed simulations using DL_POLY 4.09 and a flexible simple point charge (SPC) water potential^33^ for the 107 811-atom water box at *L*′ = 0, 1, and 2. For the *L*′ = 1 and 2 comparisons, the SPC potential was modified to include fixed dipole and quadrupole moments (based on average DL_FFLUX values). The SPC and DL_FFLUX models have in common that they are flexible. However, the flexibility in DL_FFLUX arises from *N*-body intramolecular interactions^53^ (composed of intra- and interatomic interactions). DL_FFLUX also has fully polarizable multipole moments. We mention here that kriging takes care of predicting the outcome of the polarization process (i.e., a multipole moment corresponding to a given geometry) rather than the polarization process itself (i.e., polarizability). We note that there are more recent, improved water potentials other than SPC that include polarization in various ways.^54,55^

**Figure 14:**
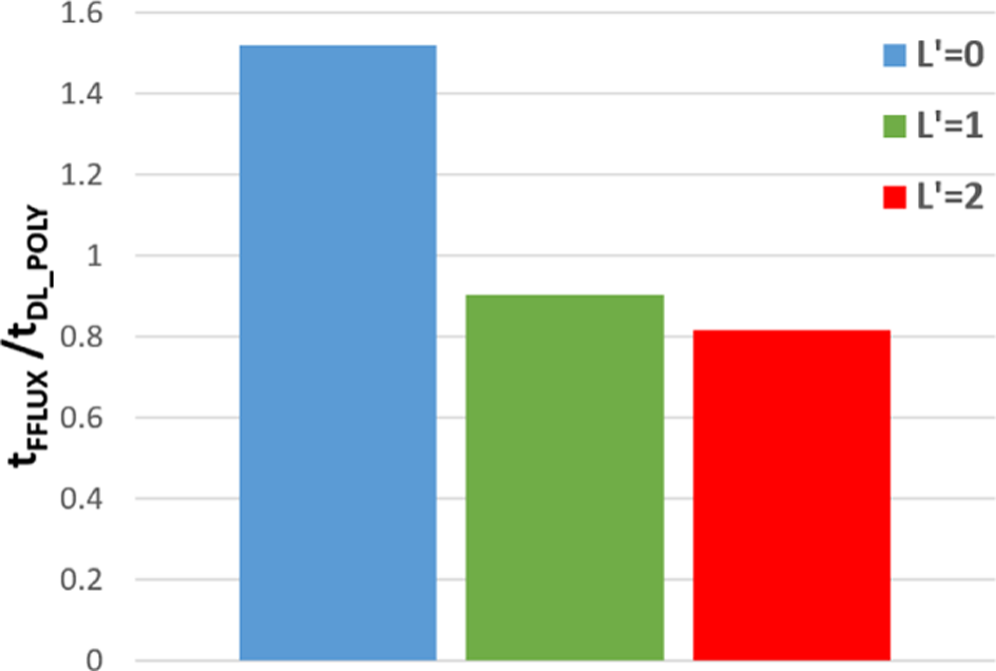
Time for a DL_FFLUX simulation divided by the time for a DL_POLY simulation with a flexible SPC water potential using the 107 811-atom water box. Averaged over all values of *N*_p_ tested.

Figure 14 shows the ratio of runtime in DL_FFLUX compared to DL_POLY, the latter using the modified SPC potential. The ratio is taken at all values of *N*_p_ (*N*_p_ = 1, 2, 4, ...) and then averaged to produce the values in Figure 14. For example, a value of 2 means that DL_FFLUX is on average 2× slower than DL_POLY. Figure 14 shows that, despite a slowdown at *L*′ = 0, DL_FFLUX is in fact quicker on average than plain DL_POLY, once higher-rank multipole moments are enabled. The speed-up with higher-rank multipole moments is largely due to some optimization of the Ewald routines in DL_FFLUX. Figure 14 is compelling evidence that DL_FFLUX can (and has been) integrated into a traditional MD package while incurring minimal extra computational overhead. This means that DL_FFLUX is now competitive in terms of speed while offering a methodology with a rigorous theoretical grounding.

**Figure 15:**
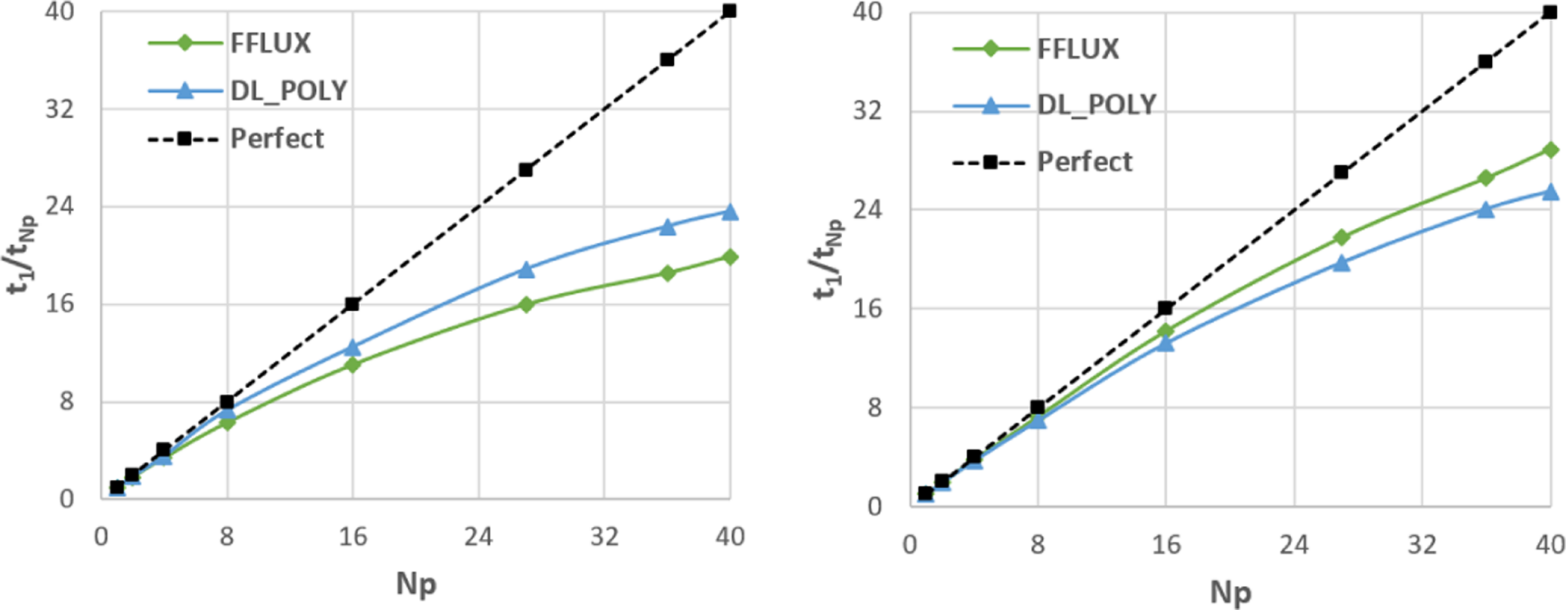
Speed-up, relative to serial, of DL_FFLUX and DL_POLY routines at *L*′ = 0 (left) and *L*′ = 2 (right).

The performance of DL_FFLUX relative to DL_POLY can be analyzed further by breaking the code into the DL_FFLUX and DL_POLY components. This is done by profiling the code and comparing the performance of the DL_FFLUX subroutines to the DL_POLY subroutines. This analysis allows examination of the strong scaling of the MPI in both parts of the code separately. This is shown for *L*′ = 0 and 2 for the 107 811-atom example. Figure 15 shows that in both cases, DL_FFLUX and DL_POLY scale in a similar way, i.e., the DD MPI implementations in both sets of routines are well integrated with each other. The reason that DL_FFLUX scales worse than DL_POLY in the *L*′ = 0 case is once again due to the redundant work when predicting point charges. Note that the scalability of the MPI in various versions of DL_POLY has been tested extensively in the past.^56,57^

**Figure 16:**
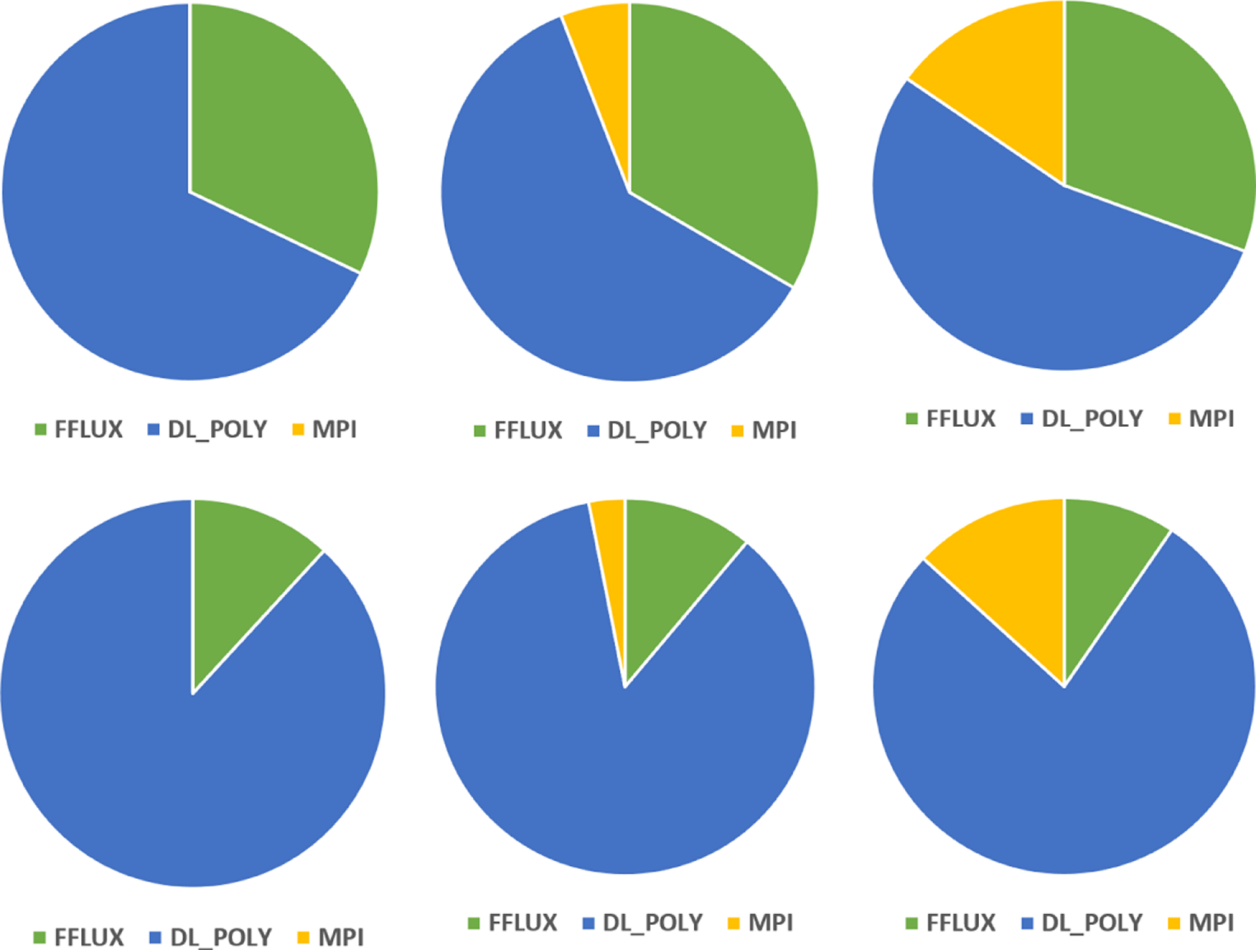
Profiles of the code at *L*′ = 0 (top row) and *L*′ = 2 (bottom row). Breakdowns are shown for *N*_p_ = 1, 8, and 36 going from left to right. The charts are not to scale, as *N*_p_ increases the total time taken decreases.

Profiling also gives us some insight into the percentage of the total runtime spent in DL_FFLUX and DL_POLY routines (as well as MPI-specific routines), which is presented in Figure 16 as pie charts. For the sake of clarity, just the profiles for *N*_p_ = 1, 8, and 36 are shown; the rest of the pie charts appear in Figure S9 as well as a more detailed “sample” breakdown in Figure S10 showing the relative timings of the top 5 most costly subroutines. Figure 16 gives a little more context to the numbers presented in Figure 14. The fraction of time spent in DL_FFLUX becomes much less important as *L*′ increases and the electrostatics starts to dominate, consistent with the trend in Figure 14. In all cases, DL_FFLUX represents a minority of the overall runtime.

Figure 16 also provides some insight into the deterioration of the strong scaling seen in Figure 10 as *N*_p_ increases. As expected, the fraction of time spent in MPI routines increases as *N*_p_ increases. This is because, as *N*_p_ increases for a fixed system size, the domain size decreases, which means that the ratio in eq 9 increases. This in turn means that each process has to communicate information about a greater fraction of the atoms in its domain. There is also the issue of the number of link cells per domain. DL_POLY has a built-in warning system that tells the user when the number of link cells per domain is smaller than 3, i.e., when the link cell method is not efficient. For the 107 811-atom system, this warning is given only when *N*_p_ = 16. Note that the warning is given for 16 and but not for 27 because, in the latter case, the space can be broken into three domains per dimension (3 × 3 × 3), whereas for *N*_p_ = 16, the decomposition is 2, 2, 4, i.e., there is an asymmetry in the dimensions leading to fewer link cells per domain in some. For the smaller systems, this warning is given considerably more frequently. In other words, there is a nonlinear relationship between the cost of communications, *t*_comms_ (the final term in eq 8), and *N*_p_. This is demonstrated more explicitly in Figure S11.

### 4.4 Best Case Timings

**Figure 17:**
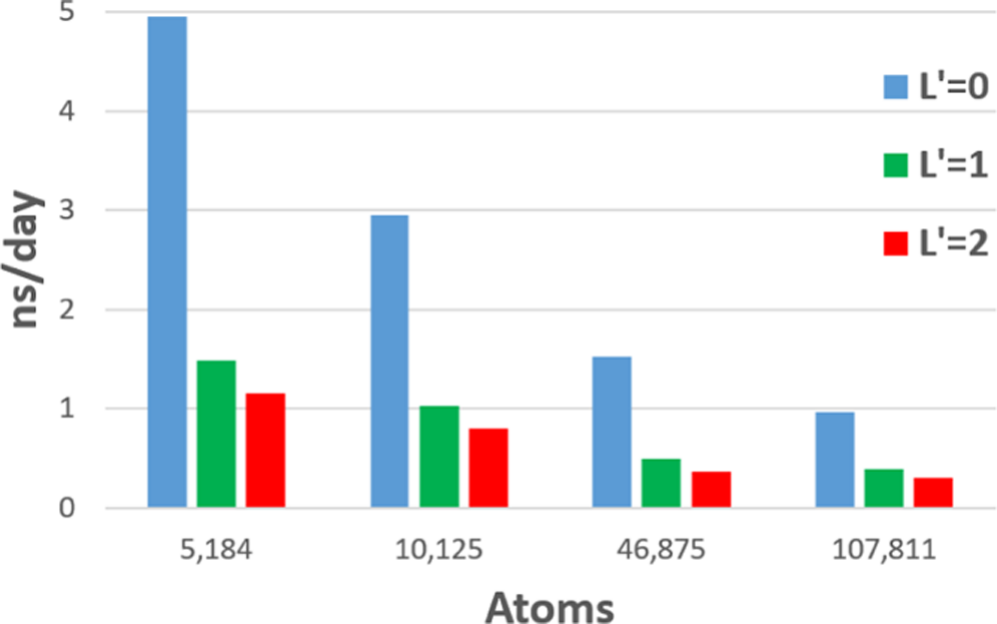
Timings at the optimum number of MPI processes for each system in nanoseconds per day.

Presented in Figure 17 are the best case timings for each of the four systems in nanoseconds per day (ns/day). This is an informative, practical metric as the behavior of water is well reproduced and studied at the scale of nanoseconds. More complex systems may require simulations on the order of tens or even hundreds of nanoseconds. Between a day and a week is a realistic time frame for a simulation. It is therefore clear from Figure 17 that DL_FFLUX is now capable of studying the behavior of systems up to approximately 10^5^ atoms in a reasonable time frame.

## 5 Conclusions

The advance presented here is that of feasibility. The rigorous methodology of FFLUX based on quantum chemical topology has already been shown to be accurate for small systems. However, the practicality of applying the method to large-scale molecular dynamics has been hampered by computational cost. The newly optimized and parallelized DL_FFLUX suffers from no such issues. DL_FFLUX can now comfortably and competitively operate on systems composed of up to 10^5^ atoms. With flexible molecules and high-rank long-range electrostatics based on polarizable atomic multipole moments, DL_FFLUX is now poised to tackle problems of real-world significance accurately and in reasonable time frames. We have also demonstrated that DL_FFLUX is a lightweight add-on to the standard DL_POLY package, incurring little computational overhead. DL_FFLUX is also well integrated into the existing DL_POLY MPI, preserving the quality of the scaling with respect to number of processes.

## Supporting Information Available

Runtime as a function of the number of atoms for varying *L*′ and *N*_p_ values (Figures S1–S4); timings (Table S1); timings as a function of *L*′ and the number of atoms (Figure S5); relative speed-ups as a function of *L*′ and an increasing number of atoms (Figures S6–S8); pie charts (Figures S9 and S10); and fraction of total runtime spent in MPI communications as a function of *N*_p_ (Figure S11) (PDF)
