# Supplementary material for: DL_FFLUX: A Parallel, Quantum Chemical Topology Force Field
Source: J Chem Theory Comput. 2021 Oct 7;17(11):7043–55. doi: 10.1021/acs.jctc.1c00595 (PMC8582247; doi:10.1021/acs.jctc.1c00595)
Supplement: Supplementary file 1 — ct1c00595_si_001.pdf [file ct1c00595_si_001.pdf]

Supporting Information

FFLUX: a parallel, quantum chemical topology force field

Benjamin C. B. Symons, Michael Bane and Paul L. A. Popelier

1. Weak scaling

Weak scaling is the scaling of runtime with problem size at fixed N_p. It is useful to examine how the run time scales as a function of system size for a given value of the number of processes N_p. This relationship gives an indication of how feasible it will be to study systems larger than the ones studied here. Figures S1 to S4 show the relationships for N_p=1,2,4 and 8 respectively, with a range extending to more than 100,000 atoms. These Figures show that the general trend is that the larger N_p, the lower the gradient of the line. Straight line fits are given, which demonstrate that the scaling is linear to a good approximation.

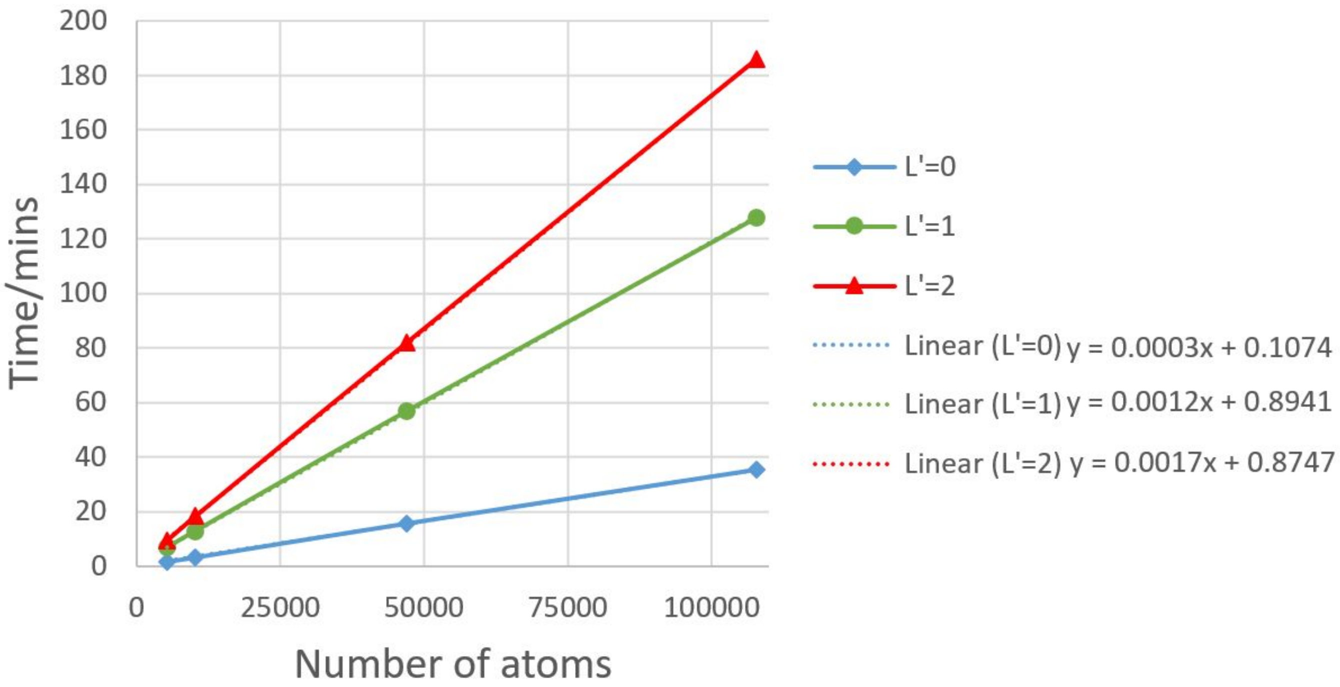

Figure S1. Weak scaling at N_p=1.

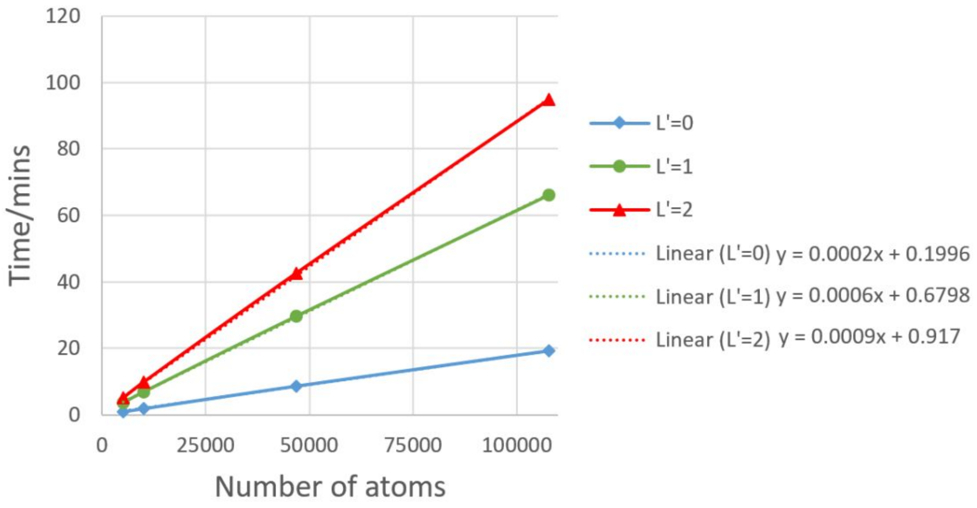

Figure S2. Weak scaling at N_p=2.

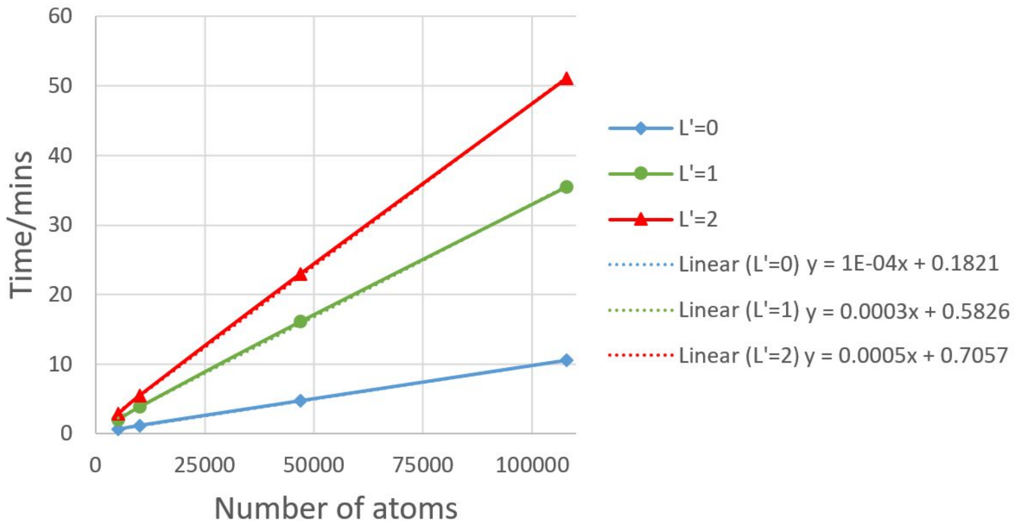

Figure S3. Weak scaling at N_p=4.

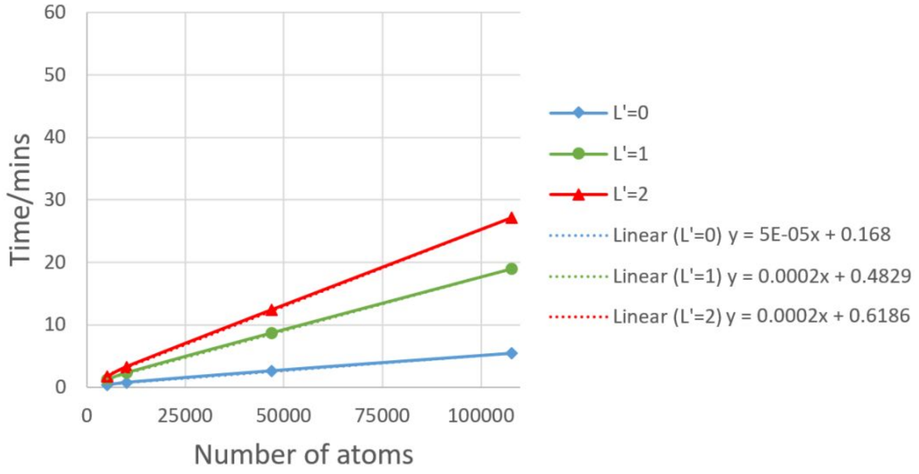

Figure S4. Weak scaling at N_p=8.

2. Cut-off radius

The effect of changing the cut-off radius on timings and scaling is interesting. All tests in the main paper were conducted with a 9 Å cut-off for both electrostatics and van der Waals interactions. However, tests were also carried out with 7 Å and 11 Å cut-off radii for the 107,811 atom system. As expected, the smaller the cut-off radius, the less time taken.

Table S1 shows that the increase in computational cost to go from 7 Å to 9 Å is relatively stable across values of L', giving an average slowdown of about 1.3 times. The average slowdown across all values of L' to go from 9 Å to 11 Å is similar at about 1.26 times. In both cases the change is modest suggesting that using a larger cut-off radius does not drastically affect the feasibility of performing a given simulation.

	**7 Å**	**9 Å**	**11 Å**
L'=0	1.59	2.21	2.91
L'=1	5.61	7.01	8.29
L'=2	7.75	9.71	12.41

Table S1. Timings (mins) for a box of water molecules with 107,811 atoms, with N_p = 27 and 1000 time steps or 1 ps.

3. Multipolar Interaction Rank L’

Another important quantity is how the code scales as a function of L’. This is particularly important as long-range electrostatics plays a crucial role in the dynamics of a system. Ideally, L’=2 is the level at which simulations will be performed. Hence the increased computational cost relative to the standard L’=0 is of great interest.

Figure S5 shows that the extra computational cost to go from L’=0 to L’=2 is approximately a factor of 4.8. Given that per atom there are 100 times more[1] electrostatic interactions to evaluate, a factor of 4.8 is a rather modest increase. It is important to mention that, at L’=2 the electrostatic routines dominate the code profile, taking approximately 66 % of the run time. At L’=0, these routines take 16 % of the run time.

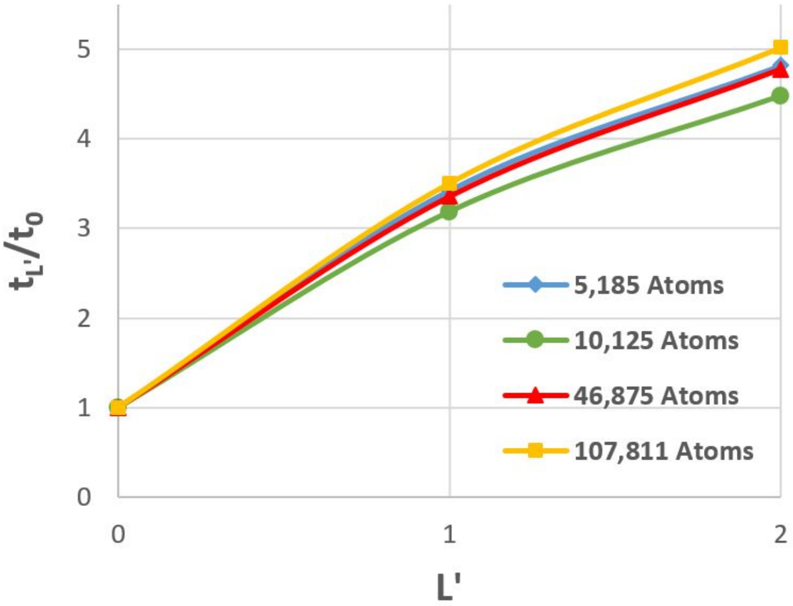

Figure S5. Scale factor relative to L'=0. Number of processes have been fixed at N_p=8. The timings t_0 and $t_{L'}$ are for L’=0 and a varying L’ value, respectively.

[1] At L’=0 there is only charge-charge but at L’=2 there is charge-charge (1), charge-dipole (3), dipole-charge (3), dipole-dipole (9=3x3), charge-quadrupole (6), quadrupole-charge (6), dipole-quadrupole (18=3x6), quadrupole-dipole (18=6x3) and quadrupole-quadrupole (36=6x6) totaling 1+3+3+9+6+6+18+18+36=100 interactions. Thus 100-fold increase in number of interactions in going from L’=0 to L’=2.

4. Scaling

Presented here are the counterparts to Figure 11 in the main paper, containing the scaling data for the remaining 3 systems.

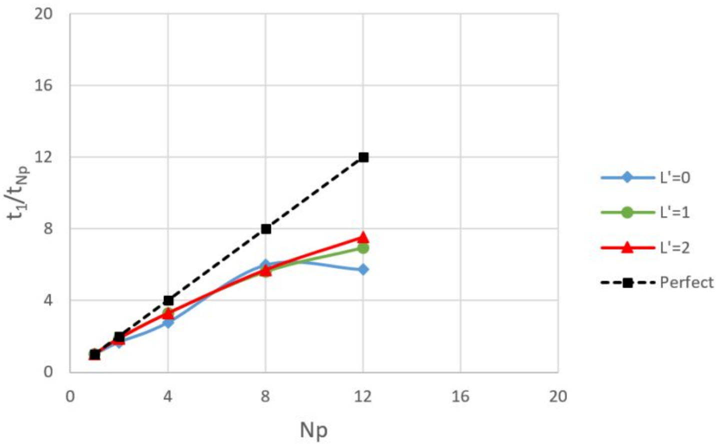

Figure S6. Speed-up relative to serial for the 5,184 atom water box.

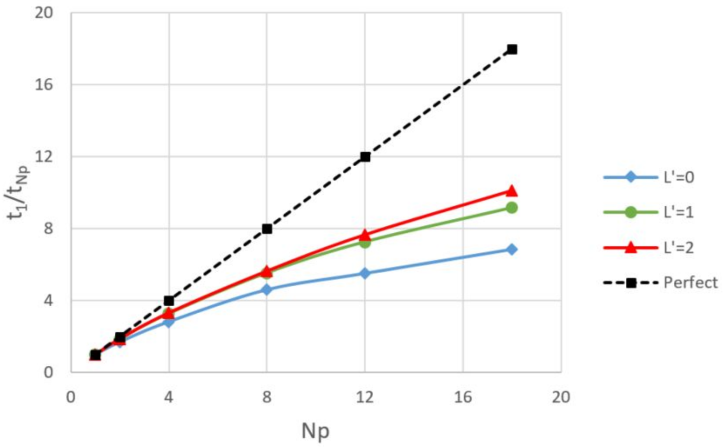

Figure S7. Speed-up relative to serial for the 10,125 atom water box.

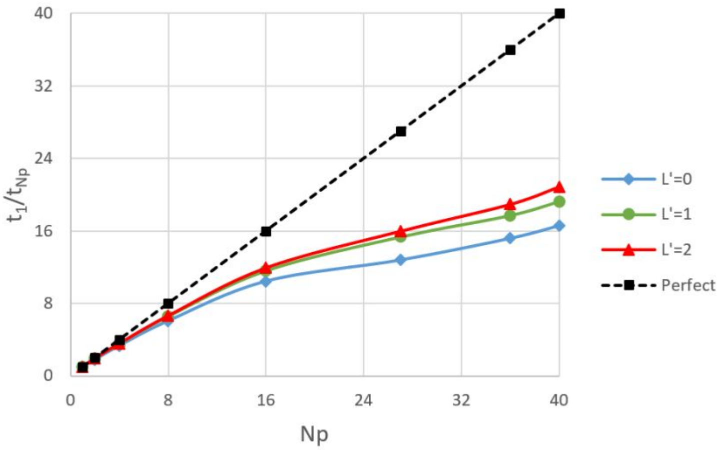

Figure S8. Speed-up relative to serial for the 46,875 atom water box.

5. Profiles

This section contains the remainder of the profiles not shown in the main paper. The trends are the same as discussed in the main paper. An example of a detailed breakdown of a profile is also shown in Figure S10. It is evident from Figure S10 that the runtime is dominated by Ewald summation subroutines (including associated subroutines, i.e. generation of B-splines in “B-spline gen”).

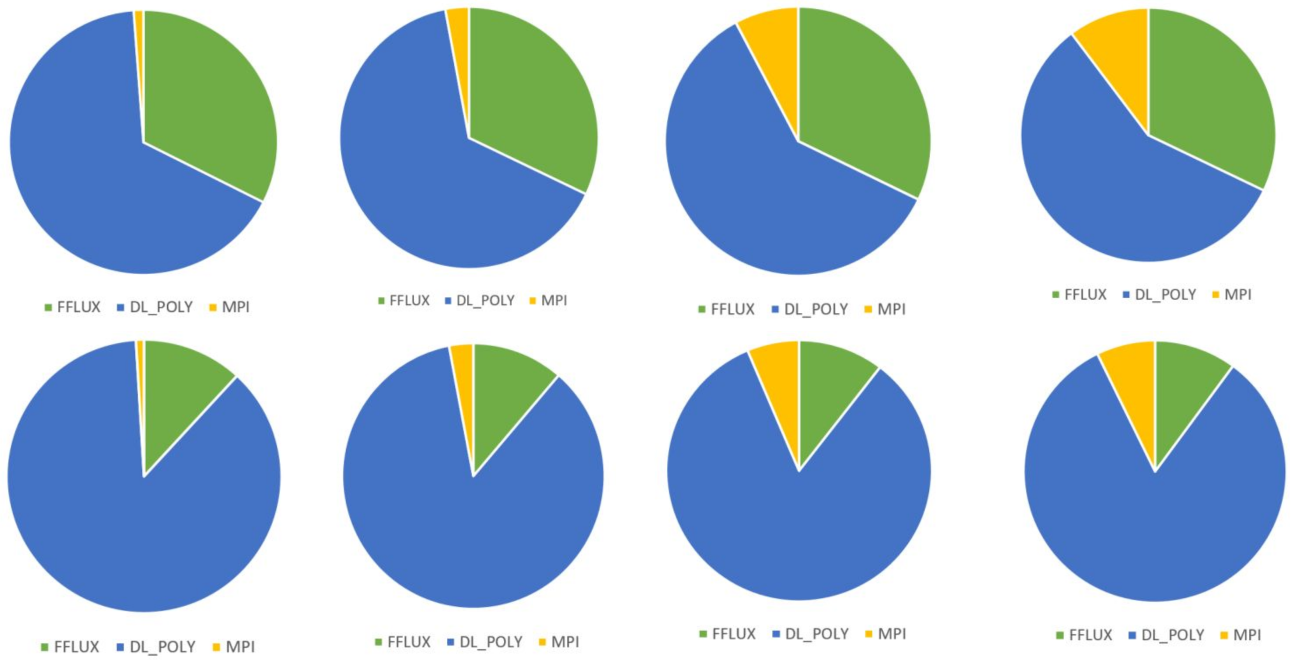

Figure S9. Remaining profiles not shown in Figure 16. L’=0 (top row) and L’=2 (bottom row) at N_p = 2, 4, 16 and 27 from left to right.

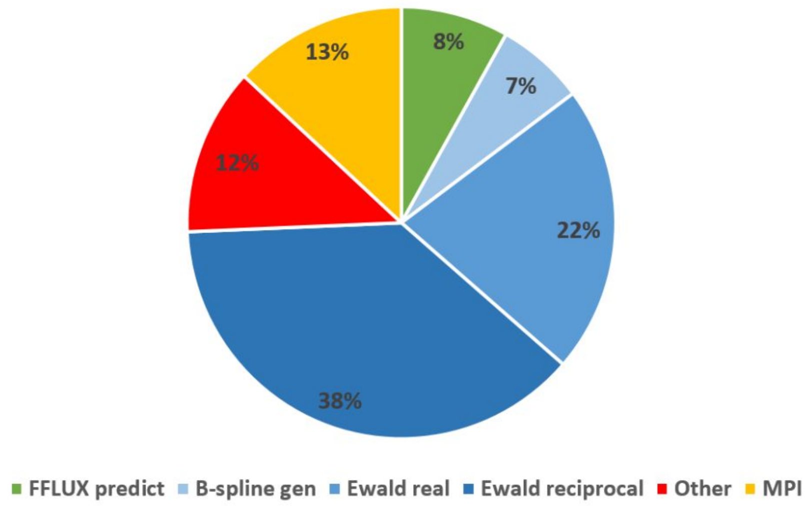

Figure S10. Breakdown of the L’=2, N_p = 36 pie chart into the top 5 most costly routines. All remaining routines are combined to form the red ‘Other’ category.

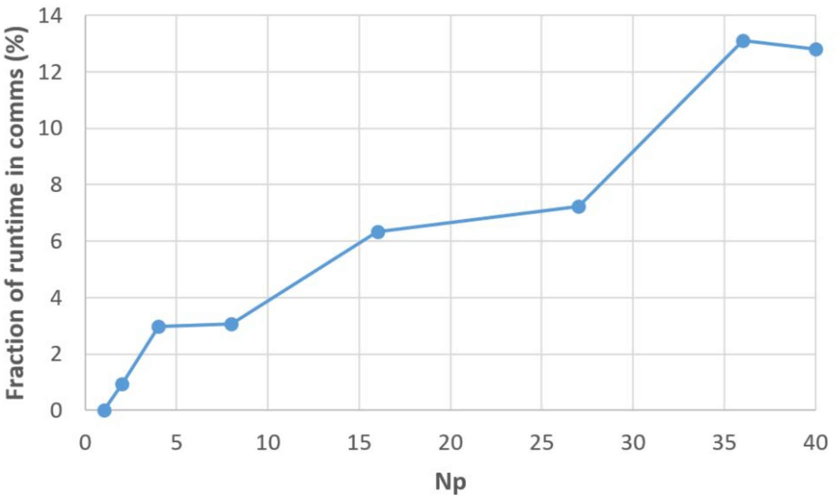

Figure S11. Fraction of the total runtime spent in MPI communications as a function of number of processes for the 107,811 atom system with L'=2.

6. Technical details on optimisation

The optimisation of FFLUX involved a large number of changes to the code, with the vast majority of subroutines being rewritten to some extent. Rather than an in-depth description of every change, only some key examples are given here.

The first example concerns the second factor in Equation 6, that is, the derivative of features with respect to global Cartesian coordinates. In the original code, the values of this factor were computed and stored in a 4D array denoted df_da. This array grew proportionally to $natms^2$ and quickly became prohibitively large, accounting for almost all of FFLUX's memory usage. A considerable amount of computational effort was also expended initialising the array to 0. Upon closer inspection this array was found to be unnecessary. Each of the array values was used once only, and so could be computed at the point of use and subsequently overwritten. As such df_da array was replaced by a single scalar variable. This resulted in a reduction in memory use of up to a factor of 10^4 for the systems studied (the larger the system, the larger the saving) and a considerable reduction in runtime.

As a second example, we mention another key issue involving the opposite problem to the df_da array above. Every atom has an associated Atomic Local Frame (ALF), which is comprised of a set of orthonormal unit vectors arranged into a matrix called the C matrix. The C matrix changes every time step and is required at several points throughout the code during a given time step. Prior to optimisation, FFLUX recomputed the C matrix every time it was needed during a given time step for every single atom. In other words, there were a huge number of redundant calculations that, when profiled, turned out to be very costly. The solution here was opposite to that of the first example. Rather than removing an array, 3 new arrays of length $natms$ were created to store each of the 3 rows of the C matrix for every atom. This increased memory use by a small amount but a massive time saving offset this increase. The tradeoff between memory use and runtime occurs repeatedly when optimising code and highlights the need for comprehensive profiling of memory usage as well as runtime.
